# Outcomes After Robot-Assisted Versus Open Pancreatoduodenectomy: A Propensity Score-Matching Analysis in a High-Volume Center (TAKUMI-7)

**DOI:** 10.3390/cancers18040602

**Published:** 2026-02-12

**Authors:** Kosei Takagi, Tomokazu Fuji, Kazuya Yasui, Yuzo Umeda, Ryuichi Yoshida, Motohiko Yamada, Takeyoshi Nishiyama, Yasuo Nagai, Atene Ito, Naohiro Okada, Shohei Yokoyama, Toshiyoshi Fujiwara

**Affiliations:** 1 Department of Gastroenterological Surgery, Okayama University Graduate School of Medicine, Dentistry, and Pharmaceutical Sciences, Okayama 700-8558, Japan; pri958hs@s.okayama-u.ac.jp (T.F.); pjyv6nvp@s.okayama-u.ac.jp (K.Y.); ryuichi-yoshida@cc.okayama-u.ac.jp (R.Y.); myamada1001@outlook.jp (M.Y.); me17063@s.okayama-u.ac.jp (T.N.); pfqz3zaq@s.okayama-u.ac.jp (Y.N.); atene0117110@yahoo.co.jp (A.I.); me421022@s.okayama-u.ac.jp (N.O.); p4v40ygp@okayama-u.ac.jp (S.Y.); toshi_f@md.okayama-u.ac.jp (T.F.); 2 Department of Hepatobiliary Pancreatic Surgery, Ehime University Graduate School of Medicine, Toon City 791-0295, Ehime, Japan; y.umeda@d9.dion.ne.jp

**Keywords:** pancreatoduodenectomy, robotic surgery, open surgery, textbook outcome

## Abstract

**Background/Objectives**: Although the safety and feasibility of robot-assisted pancreatoduodenectomy (RPD) compared to open pancreatoduodenectomy (OPD) have been reported, studies investigating the advantages of RPD remain limited. Moreover, only a few studies have investigated the effects of robotic surgery on textbook outcomes (TO). **Methods**: This single-center retrospective study included 400 patients who underwent RPD and OPD at our institution between January 2017 and December 2025. Outcomes were compared between the RPD (*n* = 162) and OPD (*n* = 238) groups using propensity score-matching (PSM) analysis. The factors associated with TO were examined. **Results**: Before PSM, significant differences were observed between the groups. PSM yielded RPD (*n* = 117) and OPD (*n* = 117) with equal preoperative factors. The RPD group demonstrated a significantly shorter operative time (402 vs. 444 min, *p* < 0.001), lesser blood loss (75 vs. 270 mL, *p* < 0.001), shorter postoperative hospital stays (13 vs. 22 days, *p* < 0.001), and fewer major complications (17.1 vs. 44.4%, *p* < 0.001), resulting in a higher TO achievement rate (76.9 vs. 52.1%, *p* = 0.001). Adjusted multivariate analyses identified robotic surgery (odds ratio 3.04, *p* < 0.001) as an independent predictor of TO. **Conclusions**: This study demonstrated that RPD was potentially superior to OPD in terms of short-term outcomes. Robotic surgery was significantly associated with TO after pancreatoduodenectomy at the expert’s hand.

## Simple Summary

The advantages of robot-assisted pancreatoduodenectomy (RPD) over open pancreatoduodenectomy (OPD) are currently unknown. In this study of 400 patients (162 with RPD and 238 with OPD), outcomes were compared using propensity score-matching (PSM) analysis. RPD demonstrated superior outcomes, including higher textbook outcome (TO) achievement rates before and after PSM. Moreover, robotic surgery was found to be significantly associated with TO after pancreatoduodenectomy. These results highlight the potential superiority of RPD over OPD in terms of short-term outcomes. Further investigation is warranted to confirm the potential long-term benefits of robotic surgeries.

## 1. Introduction

Pancreatoduodenectomy (PD) is the most commonly performed surgical procedure for resectable periampullary tumors [1]. Despite advances in surgical techniques and centralization of pancreatic surgery, postoperative morbidity remains high, with complication rates of approximately 40% and mortality rates below 5% [2,3,4]. Currently, robot-assisted PD (RPD) has been increasingly adopted to overcome the technical concerns associated with the laparoscopic approach [5,6]. Moreover, previous studies have reported the safety and feasibility of RPD compared to open PD (OPD) in high-volume centers worldwide [7,8]. However, RPD remains technically challenging and requires advanced surgical skills and knowledge of pancreatic surgery. Further evidence would help to elucidate the advantages of RPD over OPD [9].

Previous studies have focused on short-term outcomes, comparative outcomes of minimally invasive and open approaches, learning curves, and technical proficiency of RPD [10]. Among the various outcomes, the textbook outcome (TO) has been developed as a multidimensional metric for assessing the quality of pancreatic surgery [11]. However, few studies have investigated the outcomes of RPD versus OPD, focusing on TO in benign, premalignant, and malignant diseases.

This study aimed to investigate the safety and feasibility of RPD compared to OPD using propensity score-matching (PSM) analysis, as well as identify factors associated with TO, under the Training program at Okayama University for minimally invasive surgery (TAKUMI-7).

## 2. Materials and Methods

### 2.1. Study Design

This single-center retrospective study included 400 consecutive patients who underwent RPD or OPD at our institution between January 2017 and December 2025. The study was approved by the ethics committee of our institution (approval no. 2110-002) and was conducted in accordance with the Declaration of Helsinki. The need for informed consent was waived due to the retrospective nature of this study.

### 2.2. Data Collection

The following data were extracted from a prospectively maintained database: sex, age, body mass index (BMI), American Society of Anesthesiologists (ASA) physical status, comorbidities, preoperative biliary drainage, neoadjuvant chemotherapy, laboratory values, primary disease, operative variables (surgical approach, operative time, blood loss, surgeon type, conversion, and vascular reconstruction), pancreatic texture, main pancreatic duct diameter, and postoperative outcomes. Surgeons were divided into board-certified experts and surgical trainees [12]. Postoperative outcomes included length of postoperative hospital stay, mortality, reoperation, major complications [13], clinically relevant postoperative pancreatic fistula (CR-POPF) [14], post-pancreatectomy hemorrhage (PPH), bile leakage [15], and readmission within 1 month after surgery.

### 2.3. Definition of Postoperative Complications

Data on postoperative complications occurring within 1 month of surgery were recorded. Major complications were defined as grade ≥ 3 using the Clavien–Dindo classification [13]. Clinically relevant POPF (≥grade B) was evaluated using the International Study Group definition [14]. The conventional TO criteria for pancreatectomy include the absence of mortality, major complications, POPF, post-pancreatectomy hemorrhage, bile leakage, and readmission within one month after surgery [11].

### 2.4. Surgical Protocol and Selection Criteria

Surgical protocols for RPD and OPD have been described in previous publications [16,17,18]. The standard reconstruction for RPD and OPD was performed using the modified Child method [19], including pancreaticojejunostomy with the modified Blumgart method, hepaticojejunostomy, and gastrojejunostomy. For pancreaticojejunostomy anastomosis, a lost stent was placed in the RPD, and a lost or externalized stent was placed during the OPD, depending on the pancreatic duct diameter.

The concept of an enhanced recovery after surgery was first introduced in 2014 [20]. Perioperative care using an enhanced recovery after surgery protocol has been standardized for RPD and OPD. Drain management was identical between the RPD and OPD groups; drain removal was attempted after postoperative day 5 in patients with no bacterial contamination and clear drainage fluid.

RPD was introduced at our institution after officially being covered by the Japanese National Health Insurance in 2020. Our RPD protocol using the two-surgeon technique was standardized according to the Dutch Training Program (LAELAPS-3) in collaboration with the University of Pittsburgh Medical Center [21,22]. A robotic platform with a da Vinci Si or Xi system (Intuitive Surgical, Sunnyvale, CA, USA) was used.

RPD was indicated in selected patients with benign, premalignant, or malignant diseases. As RPD with vascular or other organ resection is not officially allowed by the Japanese National Health Insurance system, advanced tumors requiring vascular or other organ resection are indicated for OPD. Indications for the surgical approach were determined by a multidisciplinary team.

### 2.5. Statistical Analysis

Patient characteristics and outcomes stratified by the surgical approach were investigated. PSM analysis was performed using a logistic regression model to reduce selection bias based on potential confounding variables, including preoperative biliary drainage, neoadjuvant chemotherapy, primary disease, vascular reconstruction, and pancreatic texture. The propensity score was used for 1:1 matching with a caliper width of 0.20. Subsequently, univariate and multivariate logistic regression analyses were performed to identify the predictors of TO. Odds ratios (ORs) and 95% confidence intervals (CIs) were also determined. Values are presented as proportions for categorical data and medians (interquartile ranges [IQRs]) for continuous variables. All statistical analyses were performed using JMP software version 11 (SAS Institute, Cary, NC, USA).

## 3. Results

### 3.1. Implementation of RPD

**Figure 1:**
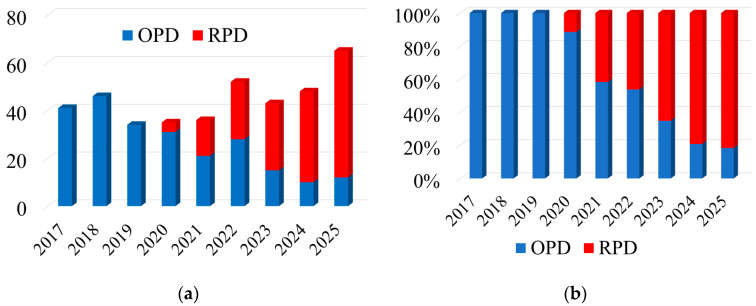
(**a**) Trends in surgical approaches of pancreatoduodenectomy over time. (**b**) Proportion of surgical approaches to pancreatoduodenectomy over time. OPD, open pancreatoduodenectomy; RPD, robot-assisted pancreatoduodenectomy.

The number of OPD and RPD conducted at our institution annually is shown in Figure 1a. Following their introduction in 2020, the number of RPDs increased steadily over time, from 11.4% in 2020 to 41.7% in 2021 and reaching 81.5% by 2025 (Figure 1b).

### 3.2. Patient Characteristics

Table 1 shows the baseline characteristics of the 400 patients. The cohort included 229 men and 171 women with a median age of 72 years (IQR, 64–77 years). Preoperative biliary drainage and neoadjuvant chemotherapy were administered in 157 and 126 patients, respectively. The laboratory values were within normal limits. The most common diagnoses were pancreatic cancer (*n* = 184, 46.0%) and benign tumors (*n* = 86, 21.5%). The cohort included 162 patients with RPD and 238 patients with OPD.

The patient characteristics regarding RPD and OPD before and after PSM are shown in Table 1. Significant differences were observed between the groups in terms of preoperative biliary drainage (*p* < 0.001), neoadjuvant chemotherapy (*p* < 0.001), and primary disease (*p* < 0.001) before PSM. After PSM, the preoperative characteristics of RPD were similar to those of OPD. In the PSM cohort, the area under the curve calculated from the receiver-operating characteristic curve was 0.787.

### 3.3. Outcomes of RPD Versus OPD

The overall outcomes are summarized in Table 2. The median operative time was 425 min (IQR: 378–489 min), with an estimated blood loss of 183 mL (IQR: 71–390 mL). Vascular reconstruction was performed in 78 (19.5%) patients. Of the 400 procedures, 330 were performed by board-certified surgeons and 70 by surgical trainees. The median postoperative hospital stay was 18 days (IQR, 13–24 days). The postoperative complications included mortality (*n* = 0, 0%), reoperation (*n* = 15, 3.8%), major complications (*n* = 128, 32.0%), CR-POPF (*n* = 60, 15.0%), PPH (*n* = 13, 3.3%), bile leakage (*n* = 11, 2.8%), and readmission (*n* = 28, 7.0%). TO was achieved in 255 (63.8%) patients. The reasons for reoperation included ileus (*n* = 3), delayed gastric emptying (*n* = 3), anastomotic leakage (*n* = 3), hematoma (*n* = 2), bile leakage (*n* = 1), wound dehiscence (*n* = 1), and others (*n* = 2). Further details on the nature of the major complications and CR-POPF included: major complications of grades 3a (*n* = 110), 3b (*n* = 14), and 4 (*n* = 4); CR-POPF of grades B (*n* = 58) and C (*n* = 2). The causes of readmission were surgical site infection (*n* = 6), cholangitis (*n* = 5), delayed gastric emptying (*n* = 4), PPH (*n* = 3), loss of appetite (*n* = 3), ascites (*n* = 2), enteritis (*n* = 2), ileus (*n* = 1), pneumonia (*n* = 1), and pancreatitis (*n* = 1).

The RPD and OPD outcomes before and after PSM are presented in Table 2. Before PSM, the RPD group had significantly shorter operative times and reduced blood loss. However, vascular reconstruction and pancreatic texture differed significantly between the groups.

In the PSM cohort, these differences were adjusted for equally. RPD was associated with a significantly shorter operative time (402 vs. 444 min, *p* < 0.001) and reduced blood loss (75 vs. 270 mL, *p* < 0.001). The RPD group had superior postoperative outcomes in terms of the length of the postoperative hospital stay (13 vs. 22 days, *p* < 0.001), major complications (17.1 vs. 44.4%, *p* < 0.001), and POPF (2.6 vs. 28.2%, *p* < 0.001), resulting in higher TO achievement (76.9 vs. 52.1%, *p* = 0.001).

### 3.4. Predictive Factors for TO

Table 3 shows the results of the univariate and multivariate analyses to identify the perioperative predictors associated with TO achievement. In univariate analyses, four variables were identified as independent factors: sex, BMI, pancreatic texture, and surgical approach. The unadjusted multivariate analyses revealed that a lower BMI (OR 2.18, 95% CI 1.23–3.86, *p* = 0.008), hard pancreas (OR 2.00, 95% CI 1.23–3.28, *p* = 0.005), and robotic surgery (OR 2.86, 95% CI 1.77–4.70, *p* < 0.001) were significantly associated with achievement of TO. After adjustment for perioperative factors, hard pancreas (OR 2.05, 95% CI 1.06–4.12, *p* = 0.034) and robotic surgery (OR 3.04, 95% CI 1.73–5.48, *p* < 0.001) were identified as independent predictors for achieving TO.

### 3.5. Outcomes of RPD Compared to Benchmark Study

Benchmark studies that have reported the outcomes of RPD are summarized in Table 4 [22,23,24,25,26,27]. Our results were comparable to those of published benchmarks, with equal surgical outcomes and a lower incidence of postoperative complications.

## 4. Discussion

The present study investigated the safety and feasibility of RPD compared with OPD in a high-volume center in Japan, including 162 RPDs and 238 OPDs, using PSM analysis. These results highlight the superiority of RPD, with significantly better short-term outcomes observed both before and after PSM. We evaluated the effects of robotic surgery on TO in patients with PD and found that robotic surgery was an independent predictor of TO after PD.

The implementation of RPD has been officially covered by the Japanese National Health Insurance since April 2020. Structured training programs, simulation training, mentorship, and centralization in high-volume centers are essential for optimizing surgical outcomes and ensuring patient safety [28]. Moreover, the median learning curve achievement point for RPD has been reported to be 36.5 cases (range: 20–80) [29]. As our surgical team participated in a nationwide training program for RPD in the Netherlands (LAELAPS-3) before the introduction of the RPD program at our institution [21,22], the influence of individual learning curves on surgical outcomes may have been limited. However, surgeons’ experience and learning curves are critical determinants of postoperative outcomes after pancreatectomy. As shown in Table 2, the RPDs were performed by only board-certified surgeons, mainly by a proctor (KT). By contrast, surgical trainees are involved in OPDs by proctoring board-certified surgeons. However, their first case was not included in the present study.

As the indications for RPD have gradually expanded over time from benign and premalignant tumors to malignant diseases, the proportion of RPD increased from 11.4% in 2020 to 81.5% in 2025 (Figure 1). Consequently, significant differences between the RPD and OPD groups were observed owing to different patient selection criteria (Table 1). Therefore, PSM was performed to balance the differences between the groups. All significant differences disappeared after PSM, with acceptable discrimination. CR-POPF is a major complication of PD, and soft pancreatic texture and small main pancreatic duct are known risk factors for POPF [30]. Because the preoperative evaluation of the pancreatic texture is difficult, this study included preoperative and intraoperative findings as matching variables. We considered that PSM using only preoperative variables, without adjusting for intraoperative variables, may mislead the interpretation of the results.

Recent meta-analyses comparing RPD and OPD demonstrated the potential advantages of RPD in terms of blood loss and postoperative outcomes, similar oncological outcomes, and the disadvantage of RPD with longer operative times [31,32]. In the present study, we found that the RPD group had superior outcomes compared to the OPD group before and after PSM, including a shorter operative time, reduced blood loss, lower incidence of complications, and shorter postoperative hospital stays, leading to a higher TO achievement rate (Table 2). Collectively, our results suggest the potential superiority of RPD over OPD with respect to short-term outcomes.

The TO rates for RPD in our study were higher than those reported in previous studies [22,33]. Structured surgical training through LAELAPS-3 likely facilitated the safe introduction of RPD at our center, which may have been associated with the higher TO rates [17]. Moreover, pancreatic texture and robotic surgery were independent predictors of TO (Table 3). To date, preoperative biliary drainage, tumor characteristics, soft pancreatic texture, small pancreatic duct, and increased blood loss have been reported as risk factors for failure to achieve TO after PD [34]. Our novel finding was that robotic surgery was associated with TO after PD. As TO achievement significantly improves survival in pancreatic cancer [35,36], robotic surgery with a higher TO achievement may contribute to improved postoperative prognosis following PD.

Although the same POPF mitigation strategies were used for RPD and OPD, the incidence of CR-POPF after RPD was notably lower in this study than in the OPD cohort and the benchmark studies cited in Table 4. Based on our previous study investigating the impact of robotic surgery on POPF in high-risk pancreaticojejunostomy, the lower incidence of CR-POPF after RPD may be attributed to precise anastomosis using the robotic platform and dedicated surgical performance [37]. Moreover, a lower drain discharge volume and incidence of positive drain fluid cultures after RPD may have led to a lower incidence of CR-POPF. In fact, a large drain volume and a high incidence of positive drain culture after OPD may cause longer drain retention, resulting in the development of CR-POPF [37].

This study has several limitations. First, this was a single-center, retrospective study with a relatively small sample size. Second, because the surgical indications for RPD and OPD differ, there may be a potential selection bias for robotic or open surgery. Although PSM analysis was performed to reduce these differences, other confounding factors may exist. Since the enrollment periods for the RPD and OPD groups were not synchronized, a chronological bias may exist. As the RPD group had a significantly later start date, this study may have exhibited a substantial selection bias. However, perioperative care has been standardized according to the enhanced recovery after surgery protocol since 2014. Therefore, it is unlikely that the institutional developments in perioperative care and the learning effects over the past three years have favored the RPD cohort in this study. Another limitation is the substantially higher proportion of procedures performed by surgical trainees in the OPD group compared to the RPD group. This imbalance may result in favorable postoperative outcomes for robotic surgery and represent a major cofounder. Finally, this study focused on the short-term outcomes of RPD and OPD, and the effect of robotic surgery on disease-specific long-term outcomes was not investigated. Similar oncological and survival outcomes have been reported between RPDs and OPDs for pancreatic cancer [26]. However, the effect of robotic surgery on long-term outcomes warrants further investigation in future studies.

## 5. Conclusions

This study demonstrated that RPD was potentially superior to OPD with regards to short-term outcomes. Robotic surgery was significantly associated with achieving TO following PD at the expert’s hand. Further studies will need to be conducted to demonstrate the potential benefits of robotic surgery in terms of long-term outcomes.

## Figures and Tables

**Table 1:** Baseline characteristics stratified by surgical approaches: overall and propensity score-matching cohort.

		Before PSM			After PSM		
Variables	Total	RPD	OPD	*p*-Value	RPD	OPD	*p*-Value
No. of patients	400	162	238		117	117	
Sex							
Men	229 (57.2)	93 (57.4)	136 (57.1)	0.958	68 (58.1)	71 (60.7)	0.690
Women	171 (42.8)	69 (42.6)	102 (42.9)		49 (41.9)	46 (39.3)	
Age, years	72 (64–77)	72 (64–77)	71 (64–77)	0.930	73 (66–77)	71 (64–77)	0.538
BMI, kg/m^2^	22.2 (20.3–24.1)	22.1 (20.6–24.0)	22.3 (20.2–24.2)	0.671	22.1 (20.6–23.6)	22.7 (20.4–24.3)	0.380
ASA score							
1–2	330 (82.5)	131 (80.9)	199 (83.6)	0.479	91 (77.8)	99 (84.6)	0.180
3–4	70 (17.5)	31 (19.1)	39 (16.4)		26 (22.2)	18 (15.4)	
Comorbidity							
Hypertension	165 (41.3)	64 (39.5)	101 (42.4)	0.559	48 (41.0)	45 (38.5)	0.689
Diabetes	102 (25.5)	41 (25.3)	61 (25.6)	0.942	33 (28.2)	29 (24.8)	0.553
Preoperative biliary drainage	157 (39.3)	46 (28.4)	111 (46.6)	<0.001	43 (36.8)	47 (40.2)	0.591
Neoadjuvant chemotherapy	126 (31.5)	26 (16.1)	100 (42.0)	<0.001	25 (21.4)	30 (25.6)	0.441
Laboratory value							
Total bilirubin, mg/dL	0.6 (0.5–0.9)	0.6 (0.5–0.8)	0.6 (0.5–0.9)	WNL	0.6 (0.5–0.8)	0.6 (0.5–0.9)	WNL
Albumin, g/dL	3.9 (3.6–4.2)	4.0 (3.7–4.3)	4.0 (3.6–4.2)	WNL	3.9 (3.6–4.2)	3.9 (3.6–4.1)	WNL
AST, U/L	21 (17–28)	20 (16–25)	22 (18–31)	WNL	21 (17–28)	24 (18–32)	WNL
ALT, U/L	19 (13–30)	17 (12–26)	20 (13–31)	WNL	17 (12–31)	21 (15–35)	WNL
Primary diseases							
Pancreatic cancer	184 (46.0)	47 (29.0)	137 (57.6)	<0.001	41 (35.0)	49 (41.9)	0.712
Bile duct cancer	52 (13.0)	24 (14.8)	28 (11.8)		22 (18.8)	22 (18.8)	
Ampullary adenocarcinoma	36 (9.0)	14 (8.6)	22 (9.2)		14 (12.0)	13 (11.1)	
Duodenal carcinoma	21 (5.3)	13 (8.0)	8 (3.4)		11 (9.4)	6 (5.1)	
Benign tumors	86 (21.5)	60 (37.0)	26 (10.9)		25 (21.4)	25 (21.4)	
Others	21 (5.3)	4 (2.5)	17 (7.1)		4 (3.4)	3 (1.7)	

Values are reported as *n* (%) or medians (interquartile range). BMI, body mass index; ASA, American Society of Anesthesiologists; PSM, propensity score matching; RPD, robot-assisted pancreatoduodenectomy; OPD, open pancreatoduodenectomy; AST, aspartate aminotransferase; ALT, alanine aminotransferase; WNL, within normal limits.

**Table 2:** Outcomes stratified by surgical approaches: Overall and propensity score-matching cohort.

		Before PSM			After PSM		
Variables	Total	RPD	OPD	*p*-Value	RPD	OPD	*p*-Value
No. of patients	400	162	238		117	117	
*Operative factors*							
Operative time, min	425 (378–489)	403 (355–446)	454 (396–511)	<0.001	402 (359–454)	444 (393–500)	<0.001
Blood loss, mL	183 (71–390)	75 (18–150)	325 (150–510)	<0.001	75 (20–165)	270 (130–445)	<0.001
Surgeon type							
Board-certified surgeon	330 (82.5)	162 (100)	168 (70.6)	<0.001	117 (100)	43 (36.8)	<0.001
Surgical trainee	70 (17.5)	0 (0)	70 (29.4)		0 (0)	74 (63.2)	
Conversion to open surgery	5	5 (3.1)	-	-	5 (4.3)	-	-
Vascular reconstruction	78 (19.5)	1 (0.6)	77 (32.4)	<0.001	1 (0.9)	1 (0.9)	1.00
Pancreatic texture							
Soft	232 (58.0)	122 (75.3)	110 (46.2)	<0.001	79 (67.5)	80 (68.4)	0.915
Hard	145 (36.3)	36 (22.2)	109 (45.8)		34 (29.1)	32 (27.4)	
Unavailable	23 (5.7)	4 (2.5)	19 (8.0)		4 (3.4)	5 (4.3)	
MPD diameter, mm	3 (2–5)	3 (2–4)	3 (2–5)	0.048	3 (2–4)	3 (2–4)	0.248
*Postoperative factors*							
Postoperative hospital stay, days	18 (13–24)	13 (9–16)	22 (17–29)	<0.001	13 (10–16)	22 (18–30)	<0.001
Mortality	0 (0)	0 (0)	0 (0)	-	0 (0)	0 (0)	-
Reoperation	15 (3.8)	7 (4.3)	8 (3.4)	0.622	4 (3.4)	4 (3.4)	1.00
Major complications	128 (32.0)	33 (20.4)	95 (39.9)	<0.001	20 (17.1)	52 (44.4)	<0.001
Grade 3a	110 (85.9)	26	84		16	47	
Grade 3b	14 (10.9)	6	8		4	4	
Grade 4	4 (3.1)	1	3		0	1	
CR-POPF (≥grade B)	60 (15.0)	6 (3.7)	54 (22.7)	<0.001	3 (2.6)	33 (28.2)	<0.001
Grade B	58 (96.7)	5	53		3	32	
Grade C	2 (3.3)	1	1		0	1	
PPH	13 (3.3)	3 (1.9)	10 (4.2)	0.18	2 (1.7)	7 (6.0)	0.08
Bile leakage	11 (2.8)	2 (1.2)	9 (3.8)	0.107	2 (1.7)	7 (6.0)	0.081
Readmission	28 (7.0)	16 (9.9)	12 (5.0)	0.066	12 (10.3)	4 (3.4)	0.034
Textbook outcome	255 (63.8)	122 (75.3)	132 (55.9)	<0.001	90 (76.9)	61 (52.1)	0.001

Values are reported as *n* (%) or medians (interquartile range). MPD, main pancreatic duct; CR-POPF, clinically relevant postoperative pancreatic fistula; PPH, postpancreatectomy hemorrhage; PSM, propensity score matching; RPD, robot-assisted pancreatoduodenectomy; OPD, open pancreatoduodenectomy.

**Table 3:** Predictors associated with achievement of textbook outcomes.

Variables	Univariate Analysis			Multivariable Analysis (Unadjusted)			Multivariable Analysis (Adjusted)		
	OR	95% CI	*p*-Value	OR	95% CI	*p*-Value	OR	95% CI	*p*-Value
Age: ≥75 years (vs. <75)	1.24	0.81–1.91	0.322						
Sex: women (vs. men)	1.56	1.03–2.39	0.035	1.53	0.97–2.42	0.067	1.41	0.79–2.58	0.248
BMI: <25 (vs. ≥25)	2.13	1.24–3.67	0.006	2.18	1.23–3.86	0.008	1.86	0.86–4.07	0.116
ASA: 1–2 (vs. 3–4)	1.21	0.71–2.05	0.475						
Preoperative biliary drainage: absence (vs. presence)	1.38	0.91–2.09	0.132						
Neoadjuvant chemotherapy: presence (vs. absence)	1.27	0.82–1.99	0.293						
Hypertension: absence (vs. presence)	1.15	0.76–1.74	0.501						
Diabetes: presence (vs. absence)	1.51	0.94–2.48	0.093						
Benign diseases (vs. malignant)	1.51	0.93–2.50	0.093						
Hard pancreas (vs. soft pancreas)	1.58	1.02–2.48	0.041	2.00	1.23–3.28	0.005	2.05	1.06–4.12	0.034
Robotic surgery (vs. open surgery)	2.41	1.56–3.76	<0.001	2.86	1.77–4.70	<0.001	3.04	1.73–5.48	<0.001

BMI, body mass index; ASA, American Society of Anesthesiologists; OR, odds ratio; CI, confidence interval.

**Table 4:** A summary of studies reporting short-term outcomes of RPD.

Authors	Our Study	Zwart et al. [22]	Zureikat et al. [23]	Lee et al. [24]	Vining et al. [25]	Liu et al. [26]	Nakamura et al. [27]
Study design	Single-center (OUH)	Multicenter (LAELAPS-3)	Single-center (UPMC)	Single-center (SNUH)	Multicenter	Multicenter	Multicenter
Country	Japan	The Netherlands	USA	Republic of Korea	USA	China	Japan
Year	2026	2023	2021	2024	2021	2023	2025
Sample size	162	635	500	630	495	1032	425
Operative time, min	403 (355–446)	395 (341−465)	415 ± 107	329 ± 81	430 ± 122	265 (240–304)	617 (456–834) *
Blood loss, mL	75 (18–150)	200 (100–450)	250 (150–400)	493 ± 665	-	190 (150–240)	160 (30–558) *
Conversion	3.1%	6.6%	5.2%	6.7%	13.5%	3.1%	3.8%
CR-POPF	3.7%	26.9%	7.8%	10.6%	11.9%	10.2%	22.1%
Bile leakage	1.2%	8.0%	-		-	6.1%	-
Postoperative hospital stay, days	13 (9–16)	11 (7–19)	8 (6–11)	11.2 ± 17.6	7 (6–9)	12 (9–16)	20 (12–41) *
Mortality	0%	3.5%	3.0% (90 d)	1.4% (90 d)	1.6% (30 d)	3.0% (90 d)	0.5% (90 d)
Readmission	9.9% (30 d)	22.8% (30 d)	35.5% (90 d)	6.5% (30 d)	24.2% (30 d)	6.6% (90 d)	-
Textbook outcome	75.3%	68.3%	-	-	-	-	-

Values are reported as median (interquartile range), * median with a range (10–90 percentile), or mean  ±  standard deviation. USA, United States of America; OUH, Okayama University Hospital; UPMC, University of Pittsburgh Medical Center; SNUH, Seoul National University Hospital; CR-POPF, clinically relevant postoperative pancreatic fistula.

## Data Availability

The original contributions of this study are included in this article. Further inquiries can be directed to the corresponding author.

## Abbreviations

The following abbreviations are used in this manuscript:

RPD	Robot-assisted pancreatoduodenectomy
OPD	Open pancreatoduodenectomy
PD	Pancreatoduodenectomy
TO	Textbook outcome
PSM	Propensity score matching
BMI	Body mass index
ASA	American Society of Anesthesiologists
AST	Aspartate aminotransferase
ALT	Alanine aminotransferase
CR-POPF	Clinically relevant postoperative pancreatic fistula
PPH	Post-pancreatectomy hemorrhage
CI	Confidence interval
OR	Odds ratio
MPD	Main pancreatic duct
